# Biodegradable PEG-PCL Nanoparticles for Co-delivery of MUC1 Inhibitor and Doxorubicin for the Confinement of Triple-Negative Breast Cancer

**DOI:** 10.1007/s10924-022-02654-4

**Published:** 2022-11-11

**Authors:** Akanksha Behl, Subhash Solanki, Shravan K. Paswan, Tirtha K. Datta, Adesh K. Saini, Reena V. Saini, Virinder S. Parmar, Vijay Kumar Thakur, Shashwat Malhotra, Anil K. Chhillar

**Affiliations:** 1 Centre for Biotechnology, M.D. University, Rohtak, Haryana 124 001 India; 2 Animal Biotechnology Centre, ICAR-National Dairy Research Institute, Karnal, Haryana 132 001 India; 3 Pharmacology Division, National Botanical Research Institute (CSIR-NBRI), Lucknow, Uttar Pradesh 226 001 India; 4 Central Research Cell and Department of Biotechnology, MMEC, Maharishi Markandeshwar Deemed University, Mullana, Ambala, Haryana 133 207 India; 5 Nanoscience Department, CUNY Graduate Center and Department of Chemistry & Biochemistry, City College, The City University of New York, 160 Convent Avenue, New York, NY 10031 USA; 6 Institute of Click Chemistry Research and Studies, Amity University, Noida, Uttar Pradesh 201 303 India; 7 Biorefining and Advanced Materials Research Center, Scotland’s Rural College (SRUC), Kings Buildings, West Mains Road, Edinburgh, EH9 3JG UK; 8 School of Engineering, University of Petroleum and Energy Studies (UPES), Dehradun, Uttarakhand 248007 India; 9 Centre for Research and Development, Chandigarh University, Mohali, Punjab 140413 India; 10 Department of Chemistry, Kirori Mal College, Delhi, 110 007 India

**Keywords:** PEG-PCL, Drug delivery system, Triple-negative breast cancer, Doxorubicin, MUC1 inhibitor, Bcl-xL

## Abstract

Combating triple-negative breast cancer (TNBC) is still a problem, despite the development of numerous drug delivery approaches. Mucin1 (MUC1), a glycoprotein linked to chemo-resistance and progressive malignancy, is unregulated in TNBC. GO-201, a MUC1 peptide inhibitor that impairs MUC1 activity, promotes necrotic cell death by binding to the MUC1-C unit. The current study deals with the synthesis and development of a novel nano-formulation (DM-PEG-PCL NPs) comprising of polyethylene glycol-polycaprolactone (PEG-PCL) polymer loaded with MUC1 inhibitor and an effective anticancer drug, doxorubicin (DOX). The DOX and MUC1 loaded nanoparticles were fully characterized, and their different physicochemical properties, viz*.* size, shape, surface charge, entrapment efficiencies, release behavior, etc., were determined. With IC_50_ values of 5.8 and 2.4 nm on breast cancer cell lines, accordingly, and a combination index (CI) of < 1.0, DM-PEG-PCL NPs displayed enhanced toxicity towards breast cancer cells (MCF-7 and MDA-MB-231) than DOX-PEG-PCL and MUC1i-PEG-PCL nanoparticles. Fluorescence microscopy analysis revealed DOX localization in the nucleus and MUC1 inhibitor in the mitochondria.

Further, DM-PEG-PCL NPs treated breast cancer cells showed increased mitochondrial damage with enhancement in caspase-3 expression and reduction in Bcl-2 expression.In vivo evaluation using Ehrlich Ascites Carcinoma bearing mice explicitly stated that DM-PEG-PCL NPs therapy minimized tumor growth relative to control treatment. Further, acute toxicity studies did not reveal any adverse effects on organs and their functions, as no mortalities were observed.

The current research reports for the first time the synergistic approach of combination entrapment of a clinical chemotherapeutic (DOX) and an anticancer peptide (MUC1 inhibitor) encased in a diblock PEG-PCL copolymer. Incorporating both DOX and MUC1 inhibitors in PEG-PCL NPs in the designed nanoformulation has provided chances and insights for treating triple-negative breast tumors. Our controlled delivery technology is biodegradable, non-toxic, and anti-multidrug-resistant. In addition, this tailored smart nanoformulation has been particularly effective in the therapy of triple-negative breast cancer.

**Supplementary Information:**

The online version contains supplementary material available at 10.1007/s10924-022-02654-4.

## Introduction

TNBC differs from other types of invasive breast cancer in that it multiplies and transmits speedily. With a dismal prognosis, it has fewer therapy options. TNBC represents 15–25% of all breast cancer incidences, and it is associated with an increased risk of localized and distal recurrence and metastatic than other malignant tumors [1–3]. Also, it is associated with a high mortality rate. Currently, effective treatments for women with TNBC are very few. Therefore, it is critical to discover and develop essential features that promote tumor onset and metastasis during breast cancer for developing innovative targeted therapy against them.

Mucins comprise glycoproteins containing a proline-threonine-serine (PTS)-rich amino acid sequence and a tandem repeat region that is extensively O-glycosylated. The human mucin (MUC) family includes MUC1 to MUC21, which are predominantly articulated on the mucosal borders of the duct endothelium. Mucins are essential in smoothing and safeguarding the epidermis of ducts, and chemical sensors, and regulating the molecular structure of the proximal cell membrane during homeostasis [4]. Mucins are believed to play a crucial role in advancing several malignancies in which their production is disrupted [5–10]. MUC1 interacts with and helps boost PI3K/AKT, ERK, and receptor tyrosine kinases (RTKs) to promote breast cancer progression [11]. On the cell surface, MUC1-C interacts with EGFR, the signaling pathway, and other receptor tyrosine kinases, triggering the PI3 and MEK pathways. MUC1-C can also get into the nucleus, where it activates the Wnt/-catenin, STAT, and NF-B RelA signaling pathways [6]. These studies demonstrated that MUC1-C is a critical target and laid the framework for the development of drugs and bioactive components that inhibit MUC1-C expression [12]. Over 90% of early TNBC tumors have high levels of MUC1, which can be attributed to genetic alterations and transcriptional abnormalities. Furthermore, MUC1 overexpression has also been linked to chemo-resistance in breast cancer [13, 14]. GO-201 peptide is a MUC1 inhibitor that inhibits MUC1 function, slows cell growth, and causes necrotic cell death by adhering to the MUC1-CQC motif at amino acids 87–89 of the MUC1-C subunit. MUC1 inhibitor (GO-201) preferentially triggers cancer cell death by blocking transcriptional activation of Bcl-xL, an anti-apoptotic protein [15].

Doxorubicin (DOX) is an effective first-line therapy for a variety of malignancies. It could be used alone or with concomitant chemotherapy drugs. DOX can influence gene expression by intercalating into DNA, producing reactive oxygen species, and inactivating topoisomerase [16]. Regardless of whether one or more of these hypothesized routes are activated, DOX exhibits a cell-cycle-specific effect.DOX can also activate both p53-dependent and p53-independent apoptotic pathways [17]. However, DOX therapy suffers from long-term hazardous effects of irreversible cardiomyopathy based on the average dosage [18]. Increased amounts of reactive oxygen species in the heart resulting in apoptosis play a vital role in DOX-mediated cardiomyopathy. Nanocarriers-based smart drug delivery systems provide deeper tissue penetration and specific drug targeting, ultimately improving the safety and effectiveness of anti-cancer drugs [18, 19].

Because of its amphiphilic nature, degradability, biocompatibility, and semi-crystalline structure with such a mild glass transition temperature, poly (ethylene glycol) methyl ether-block-poly (-caprolactone) (PEG-PCL) copolymers are attractive therapeutic components. PEG-PCL copolymers are good contenders for continuous drug delivery applications due to their long-term drug release features. These unique properties are attributed to their application as anticancer drug delivery platforms in nanoparticle form. To encapsulate both doxorubicin and MUC1 inhibitors in a cocktail, we have used PEG-PCL diblock copolymer, which has an amphiphilic propensity. The most important qualities are its biodegradability and sustained release, which are ideal for delivering the drugs to their target cells [20].

Inside this research, we developed a DOX and MUC1 inhibitor-loaded PEG-PCL polymeric nanoparticle system for targeting TNBC to reduce doxorubicin drug toxicity in healthy cells and boost therapeutic effectiveness. GO-201 peptide was also incorporated inside the PEG-PCL nanocarriers along with DOX, which is known for MUC1 inhibition and helps drive the smarter targeted drug delivery system towards its active diseased site. The DOX and MUC1 loaded nanoparticles were characterized physicochemically to determine their size, shape, surface charge, entrapment efficiencies, release behavior, etc. The cytotoxicity parameters of the DOX and MUC1 loaded nanoparticles (DM-PEG-PCL NPs) were evaluated and assessed against the breast cancer cell lines MCF-7 and MDA-MB-231. Our studies have revealed much lower IC50 values (nm) for DOX and MUC1i-loaded nanoparticles (DM-PEG-PCL NPs) as compared to DOX-PEG-PCL (nanoparticles with DOX) and MUC1i-PEG-PCL (nanoparticles with MUC1i) when loaded separately and taken as controls. Localization studies for DOX and MUC1i were performed using Fluorescence microscopy. The In vivo studies were carried out using the EAT-bearing mice model, which indicated that providing DM-PEG-PCL loaded NPs reduced tumor growth as compared to negative controls. The acute toxicity profile studies have also been carried out using DM-PEG-PCL nanoparticles, and their effects on organs and mortality numbers were analyzed. This synergistic approach involving concomitant loading of DOX with the MUC1 inhibitor in PEG-PCL polymer is an innovative and novel strategy for the treatment of triple-negative breast cancer.

## Materials and Methods

### Materials

Doxorubicin (DOX), GO-201 trifluoroacetate salt (MUC1 inhibitor), Rhodamine 123 (Rh 123), Rhodamine B (RhoB), 4,6-diamidino-2-phenylindole (DAPI), Fluorescein isothiocyanate (FITC), Coumarin-6, poly ethylene glycol-5000 (PEG-5000), caprolactone, Tin (II) 2-ethylhexanoate, 3-(4,5-Dimethylthiazol-2-yl)-2,5-Diphenyltetrazolium Bromide (MTT), Dimethylsulfoxide (DMSO), Bradford reagent, chemiluminescence reagent purchased from Sigma aldrich (Merck, USA). Amicon ultracentrifugal filters (10 kDa), Protein estimation kit by BCA (Bicinchoninic acid) method (GeNei) were obtained from Merck Millipore (Billerica, MA, USA.) Dulbecco’s modified Eagle’s medium (DMEM), fetal bovine serum (FBS), Trypsin, Dulbecco Phosphate Buffered Saline (PBS) and penicillin–streptomycin antibiotic solution were purchased from Gibco (Life Technologies), USA. Acridine Orange (Thermo scientific), 4% Paraformaldehyde, Crystal Violet, Radio-immunoprecipitation assay (RIPA) lysis buffer, Tris-buffered saline (TBST) were obtained from Hi Media (India). Annexin V-FITC assay kit was obtained from BD Biosciences (San Jose, USA). Primary antibodies i.e. caspase-3 and Bcl-2 were procured from Sigma Aldrich (USA), β-actin and secondary antibodies were purchased from (Santa Cruz Biotechnology, USA). X-ray films were purchased from Amersham Biosciences, UK.

### Methods

#### PEG-PCL Copolymer Synthesis and Characterization

PEG (MW 5000 Da) was azeotropically distilled with toluene over 4–6 h, to eliminate moisture. Caprolactone (CL) was dehydrated through molecular sieves for 24 h. Sn(Oct)_2_ was used as a catalyst in the ring-opening polymerization of CL with PEG homopolymer to synthesize the PEG-PCL di-block copolymer. In a 10 mL round bottom flask coupled to a vacuum, 100 mg PEG and 98 mg CL were added. The reaction was carried out in a vacuum at 160 °C; the content was purified by cooling the reaction mixture and diluting it with diethyl ether. The resulting product was filtered before even being dried in a vacuum oven [21].The synthesized PEG-PCL copolymer was characterized from its ^1^HNMR (400 MHz, Brukers, USA) and FTIR (RZX, Perkin Elmer, UK) spectral data to validate the coupling of PEG with caprolactone.

#### Preparation and Characterization of DOX and MUC1 Inhibitor Loaded PEG-PCL Nanoparticles (DM-PEG-PCL NPs)

**Fig. 1:**
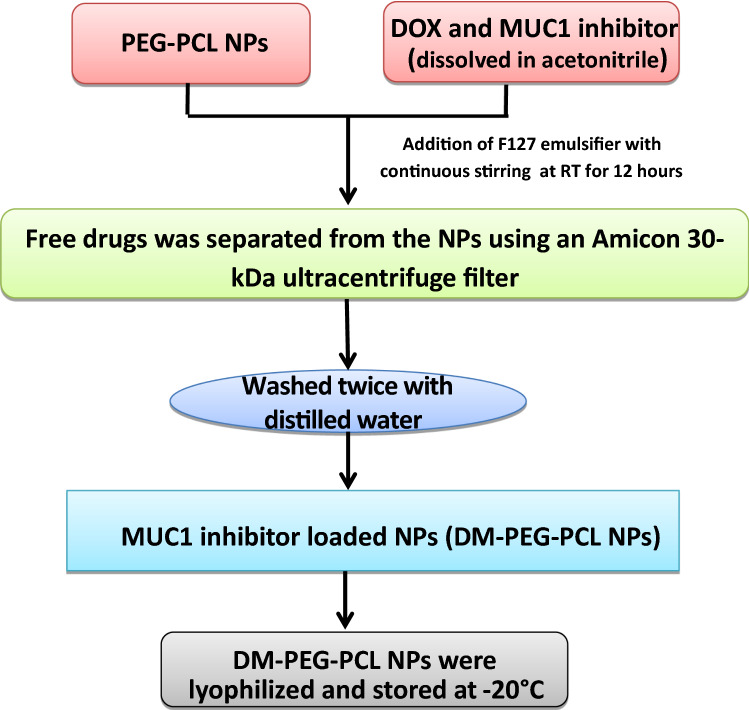
Schematic for the synthesis of DM-PEG-PCL NPs

A double emulsion evaporation method was used to load DOX, and MUC1 inhibitor in PEG-PCL nanoparticles [22, 23]; PEG-PCL copolymer was dissolved in acetonitrile under sonication, after that with the help of a syringe needle DOX and MUC1 inhibitor were added to the solution. The contents were shaken for 12 h at room temperature with 200 mg of F127 emulsifier in a 40 mL aqueous medium to enhance nanoparticle stabilization. To remove unbound drug/dye, NPs were filtered through Amicon 10 kDa ultracentrifuge filter and washed thrice. The nanoparticles were lyophilized and kept at −20 °C until required. With hydrophilic Rhodamine (Rho B) and hydrophobic Coumarin-6(C6), a similar approach was utilized to synthesize dye-loaded NPs. A BCA test has been used to determine the quantity of free MUC1 inhibitor at 595 nm in the filtrate and by using high-performance liquid chromatography, the amount of free DOX was quantified (HPLC; Perkin Elmer, USA). Figure 1 depicts the systematic synthesis of DOX and MUC1 inhibitor-loaded nanoparticles by the double emulsion evaporation method.

$$\% {\text{Encapsulation Efficiency}} = \frac{(Drug) Total -(Drug)\; Filtrate}{Drug\;Total}\times 100$$

#### DOX and MUC 1 Inhibitor (GO201) In vitro Release From Polymeric NPs

Using an ultrafiltration method, the In vitro release kinetics of DOX and MUC1 inhibitor from NPs were evaluated. Thawed drug-loaded NPs were resuspended in pH 7.4 PBS and kept at room temperature with continual shaking. At predefined intervals up to 60 days, samples were obtained and ultra-filtered using 10 kDa Amicon filters. The filtrates were removed and reconstituted with a new buffer for analyses [24]. The filtrates were evaluated for DOX at 488 nm using HPLC and for MUC1 inhibitor at 595 nm by BCA assay.

#### PEG-PCL NPs Uptake Study

MCF-7 cells (2 × 10^5^ cells/ml) were cultures on sterilized coverslips. After 24 h RhoB and coumarin-6 loaded NPs were added. The coverslips were removed and cells were rinsed with ice-cold PBS, the cells were fixed for 15 min with 4% paraformaldehyde. Before being examined under a fluorescence microscope (Olympus IX-73, Japan), the cells were stained with DAPI (1 µg/ml), then rinsed three times in ice-cold PBS. Flow cytometry (FACS, Becton Dickinson Biosciences, USA) was used to study the cellular absorption of NPs [25].

#### In vitro Cytotoxic Effect of NPs

The MTT cell viability assay was used to assess the anti-proliferative effect of NPs In vitro*.* Human luminal hormone-dependent MCF-7 and triple-negative MDA-MB-231 breast cancer cell lines were purchased from NCCS, Pune, India. The cells were grown in DMEM with 10% FBS, 100 units/mL penicillin, and 100 μg/mL streptomycin at 37 °C. In a 96-well tissue culture plate (NUNC), both cancer cell lines were seeded at a density of 10,000 cells per well and incubated for 24 h. All of the individual NPs and their combinations were dissolved in DMSO at concentrations of 0.005, 0.01, 0.03, 0.06, 0.125, 0.312, 0.625, 1.25, 2.5, 5, 10, 20, and 40 µM [26], the untreated control cultures with DMSO < 0.2%. The half-maximal inhibitory concentration (IC_50_) was calculated using Graph Pad Prism (6.0). The data is provided as a mean ± standard deviation (*n* = 3). The MTT assay was carried out as explained previously [27], and the percent cell viability and growth inhibition was calculated using the formula below.

$$\mathrm{\% cell\ viability}=\frac{Absorbance\ (Sample)}{Absorbance\ (control)}\times 100$$

$$\% cell\ growth\ inhibition=100-\%\ cell\ viability$$

#### Combination of Drugs Through Combination Index (CI) Analysis

At a 1:1 molar ratio, the potential for drug interaction between DOX NPs and MUC1 inhibitor NPs was evaluated. The following formula was used to get the CI value based on isobologram analysis:

$${\text{Combination index}}\left( {CI} \right) = \frac{{\left( {D1} \right)}}{{\left( {Dx} \right)1}} + \frac{{\left( {D2} \right)}}{{\left( {Dx} \right)2}}$$

where (Dx)1 and (Dx)2 are the individual concentrations of DOX NPs and MUC1 inhibitor NPs sufficient to inhibit cell growth by 50%, respectively, and (D)1 and (D)2 are the drug concentrations needed to inhibit cancer cell growth by 50% used together. CompuSyn Inc., USA (version 1.0) software has been used to evaluate the quantitative data, based on median effect principle proposed by Chou and Talalay [28]. The CI values suggest whether the relationship is synergistic (CI < 1), additive (CI = 1), or antagonistic (CI > 1). At a constant drug combination ratio, graphs with *fa* (fraction affected) vs CI were generated.

#### Loss Mitochondrial Membrane Potential (MMP)

Rh 123 staining has been used to determine the change in MMP (ΔΨm) as a sign of mitochondrial instability. MCF-7 cells at the density of 3 × 10^5^cells/well were seeded in 12-well plates for 24 h and then treated with DOX-PEG-PCL NPs, MUC1i-PEG-PCL NPs, and DM-PEG-PCL NPs at IC_50_ (25.7 nM, 43.3 nM, and 5.8 nM) values, respectively for 48 h. The 10 µM Rh 123 dye was incubated for 20–30 min [29]. The plates were examined by fluorescence microscopy (Olympus IX-73, Japan) at 60X magnification.

#### Clonogenic Assay In Vitro

MCF-7 cells were cultivated at a density of 2 × 10^5^ cells/ml in a 6-well tissue culture plate. The cells were treated with DOX-PEG-PCL NPs, MUC1i-PEG-PCL NPs, and DM-PEG-PCL NPs at their IC_50_ (25.7 nM, 43.3 nM, and 5.8 nM) values, respectively. After the treatment, harvest and count the cells, and re-seed at a density of 1 × 10^3^ on a 6-well plate at 37 °C for 7–10 days to form colonies. The colonies were then preset in 4% paraformaldehyde for 15 min, dyed with 0.5% crystal violet dye, and air dried before counting and photographing [30, 31].

#### Effect of NPs on Cell Migration

MCF-7 cells were grown at a density of 1 × 10^5^ cells/well in a 6-well culturing plate to develop confluent monolayers. The straight wounds were created using 200 µL pipette tips. Following washing, damaged monolayers were cultured in media with 1% FBS to inhibit cell multiplication and treated with DOX-PEG-PCL NPs, MUC1i-PEG-PCL NPs, and DM-PEG-PCL NPs at their IC_50_ (25.7 nM, 43.3 nM, and 5.8 nM) values, respectively [32]. The wound gaps were recorded after an interval of 48 and 72 h, and the % of wound/ gap closure was derived from the following formula:

$$\mathrm{\% Wound\ Closure}=1-\frac{\mathrm{wound\ area\ at }\ 0\mathrm{ h}}{\mathrm{wound\ area\ at\ }48/72\mathrm{ h}}\times 100\mathrm{\%}$$

#### DM-PEG-PCL NPs /NPs Induced Apoptosis

Annexin V + cells were analyzed using a flow cytometer to investigate apoptosis induced by DM-PEG-PCL NPs. MCF-7 cells were grown at a density of 2 × 10^5^ cells per well in a 6-well plate. The cells were treated with DOX-PEG-PCL NPs, MUC1i-PEG-PCL NPs, and DM-PEG-PCL NPs at IC_50_ (25.7 nM, 43.3 nM, and 5.8 nM) values, respectively for 48 h. Paclitaxel was taken as a positive control. The cells were rinsed 3 times with binding buffer after incubation and stained with Annexin V-conjugated with FITC, according to the manufacturer's instructions. The percentage of live, apoptotic, and necrotic cell counts was assessed using FACS (Becton Dickinson, USA), and microscopy images were acquired by using Olympus IX-73 fluorescent microscope [33].

#### Immunoblot Analysis

Transfected MCF-7 cells were cultivated at a concentration of 4 × 10^6^ cells/ml and treated with NPs for 48 h. The cells were harvested after treatment and RIPA lysis buffer was added to get the cell lysate. The lysates were used to perfoem western blot analysis. Immunoblot analysis was performed using primary Bcl-2, caspase-3, and β-actin antibodies. [34]. The protein bands were visualized and the signals were caught on X-ray films using the enhanced chemiluminescence reagent [35].

#### Acute Toxicity of the NANOPARTICLES

Acute toxicity of the NPs was evaluated in Swiss albino mice weighing 20–25 g using the up and down procedure [36]. Animals were treated with DOX-PEG-PCL NPs, MUC1i-PEG-PCL NPs, and DM-PEG-PCL NPs (2000 mg/kg body wt.). The animals were inoculated p.o. and observed for behavioral profile, neurologic profile, physical states, and mortality for 14 days. After 14 days, blood was taken from mice for the hematological assay.

#### In vivo Anti-Tumor Effective of NPs

The In vivo research followed the guidelines set forth by the Institutional Animal Ethical Committee (IAEC) (1767/RE/S/14/CPCSEA, dated August 31, 2017). Ehrlich ascites carcinoma (EAC) cells were administered intraperitoneally in Swiss albino female mice (18–22 g). These EAC cells were harvested and injected (intramuscular, 1 × 10^7^ cells) into right thigh of the Swiss albino mice (0 day). The mice were separated into six groups (n = 7) and given intravenous injections of sterile saline, DOX-PEG-PCL NPs (10 mg/kg), MUC1i-PEG-PCL NPs (10 mg/kg), DM-PEG-PCL NPs (10 mg/kg), and 5-FU (22 mg/kg, positive control) [37]. The body weights of NPs-treated mice were recorded on 13th day. After the treatment profile was completed (day 13), the tumor’s height and width were calculated with a digital vernier caliper, and the tumor weight was estimated as follows:

$$\mathrm{Tumor\ weight }\left(\mathrm{mg}\right)=\frac{Length\ \left(mm\right) \times {[Width (mm)]}^{2}}{2}$$

### Statistical Analysis

The data were presented as mean ± SD. The analysis used a sample size of at least three determinations. The collected data were analyzed using a one-way ANOVA followed by a Bonferroni post-test. The findings were statistically significant for at least *p < 0.05 at the level of significance. ImageJ was used to do the densitometric study.

## Results and Discussion

### Synthesis and Characterization of PEG–PCL Co-Polymer

The PEG-PCL copolymer was prepared using a previously described process [20]. The synthesized PEG–PCL di-block copolymers’ structure and content were investigated using ^1^HNMR and FTIR spectroscopy. Figure S1 illustrates the ^1^HNMR spectra of PEG-PCL (in CDCl_3_). Methylene protons (CH_2_) were found in PEG-PCL at concentrations of 1.5 ppm, 1.7 ppm, 2.4 ppm, and 4.12 ppm, which correspond to caprolactone methylene protons. The protons associated with the methoxy (OCH_3_) and methylene (CH_2_) groups of PEG were contained in the PEG-PCL polymer at roughly 3.48 and 3.61 ppm, respectively. Figure S2 indicates a prominent absorption band around 1724.5 cm^−1^ in the FT-IR spectra of the PEG–PCL copolymer, which ascribed to the carbonyl group (C = O) of the carboxylic ester present in the PEG–PCL polymer. The characteristic C–O–C stretching bands of the repetitive –OCH_2_CH_2_ units of PEG and the –COO– bond stretching frequency might attribute to the absorption bands at 1102.9 and 1240.25 cm^−1^. The PEG–PCL copolymer was successfully developed as entire C–H stretching bands were centered at 2958 and 2885.3 cm^−1^.

### Synthesis of PEG-PCL Nanoparticles and Their Physico-Chemical Characterization

Using a double emulsion evaporation process [22], the di-block copolymeric DOX-PEG-PCL NPs (DOX entrapped PEG-PCL polymer), MUC1i-PEG-PCL NPs (MUC1i entrapped PEG-PCL polymer), and DM-PEG-PCL NPs (both DOX and MUC1i entrapped in PEG-PCL polymer) were synthesized. Drug trapping, particle density, and, eventually, drug release properties of nanoparticles are all regulated by the drug-polymer ratio. The drug release from the nanoparticles was faster when the drug-polymer ratio is higher. The drug/polymer ratio used for the synthesis of these NPs was 1:10, which is suitable for drug loading and drug release. This might be explained by a rise in the drug/polymer ratio as the amount of drug-loaded polymer grows, implying that more therapeutics are liberated per unit area of accessible polymer matrix interface. Table 1 displays the average particle sizes, zeta potentials, entrapment efficiencies, and polydispersity index (PDI) of the synthesized NPs. The average particle sizes for DOX-PEG-PCL NPs, MUC1i-PEG-PCL NPs, and DM-PEG-PCL NPs as determined by DLS were 94, 95, and 175 nM, respectively, with low polydispersities (PDI), indicating that single drug-loaded NPs (DOX-PEG-PCL and MC1i-PEG-PCL) are smaller in sizes. Due to two drugs entrapped in the core of NPs, the size of cocktail drug-loaded NPs (DM-PEG-PCL) is larger compared to single drug-loaded NPs. The synthesized nanoparticles were homogeneous in size, as evidenced by their low PDI values (Table 1). The zeta potentials (surface charge) of DOX-PEG-PCL NPs, MUC1i-PEG-PCL NPs, and DM-PEG-PCL NPs were found to be −22.33, −20.26, and −1.6 mV’s, respectively (Table 1). Negatively charged nanoparticles are more likely to be rejected by the negatively charged cell membranes [38]. Our synthesized DM-PEG-PCL NPs showed very low negative zeta potential values (close to neutral) compared to other synthesized NPs. The low negative charge on these developed nanoparticles (DM-PEG-PCL NPs) would be useful in tumor accumulation and sustained blood circulation for breast cancer treatment [39].

**Table 1:** Physico-chemical characteristics of di-block PEG-PCL NPs

Nanoparticles (NPs)	Size (by DLS) nM)	Zeta Potential (in mV)	DOX:MUC1inhibitor (w/w)	Drug/Polymer ratio	%EE DOX	%EE MUC1 inhibitor	PDI
PEG-PCL NPs	64	−13.5					0.079
MUC1i-PEG-PCL NPs	95	−20.2	0:1	1:10	–	93.3	0.017
DOX-PEG-PCL NPs	94	−22.3	1:0	1:10	85.5	–	0.088
DM-PEG-PCL NPs	175	−1.6	1:1	1:10	86.8	89.2	0.010

To ascertain the morphology (shape) of the synthesized nanoparticles, transmission electron microscopy (TEM) measurements were performed on aqueous sample solutions of PEG-PCL NPs, MUC1i-PEG-PCL NPs, DOX-PEG-PCL NPs and DM-PEG-PCL NPs at a general concentration of 1 mg/ml. From the Figure S3, it can be inferred that all the four synthesized nanoparticles viz. PEG-PCL NPs, MUC1i-PEG-PCL NPs, DOX-PEG-PCL NPs, and DM-PEG-PCL NPs showed a tendency to form spherical particles in the range of 20–180 nM.

When TEM data were compared to DLS measurements, the average diameter (size) of the produced nanoparticles was found to be significantly lower. Figure S4 depicts the DLS of DM-PEG-PCL NPs. The larger particle size distribution in DLS was attributed to the PEG-PCL hydrodynamic shell, which could be influenced by composition (bigger coordination sphere of PEG-PCL nanoparticles). Even a little proportion of bigger particles (1–2% by volume) can drastically alter the particle size distribution.

Using *UV–Vis* spectroscopy, the loading efficiencies of DOX and MUC 1 inhibitor separately (i.e., for DOX-PEG-PCL NPs and MUC1i-PEG-PCL NPs) in the PEG-PCL NPs were determined and found to be 85.5% and 93.3%, respectively, (Table 1) indicating that enough drug was encapsulated in our nanoparticles. The entrapment efficiencies of DOX and MUC1 inhibitor together in di-block PEG-PCL NPs (i.e., in DM-PEG-PCL NPs) were 86.8% and 89.2%, respectively. PEG-PCL (Mn 30,000; PDI ≤ 1.6) is amphipathic, with PEG forming the hydrophilic exoskeleton (shell) and PCL constituting the central hydrophobic core. The synthesized PEG-PCL block co-polymer forms spherical nanoaggregates with a critical micelle concentration (CMC) of 4.8 × 10^−3^ mg/ml [40]. The lower CMC suggested that they were more thermodynamically stable, which was beneficial for infusing them into body fluids. This occurred when PCL units were integrated into the block copolymer chain [20]. This feature of the di-block co-polymer PEG-PCL encapsulates both hydrophilic and hydrophobic drugs efficiently, even at lower concentrations. The hydrophobic portion of this copolymer enables hydrophobic drugs to be entrapped in the nanoparticle core. The use of the conventional anti-cancer drug DOX is restricted due to its toxicity of free drug for healthy tissues, low solubility, and inherent MDR effects. To solve the limitations of toxicity, MDR, and increased selectivity for cancerous cells, the hydrophobic DOX was entrapped in the core of these PEG-PCL nanoparticles.

**Fig. 2:**
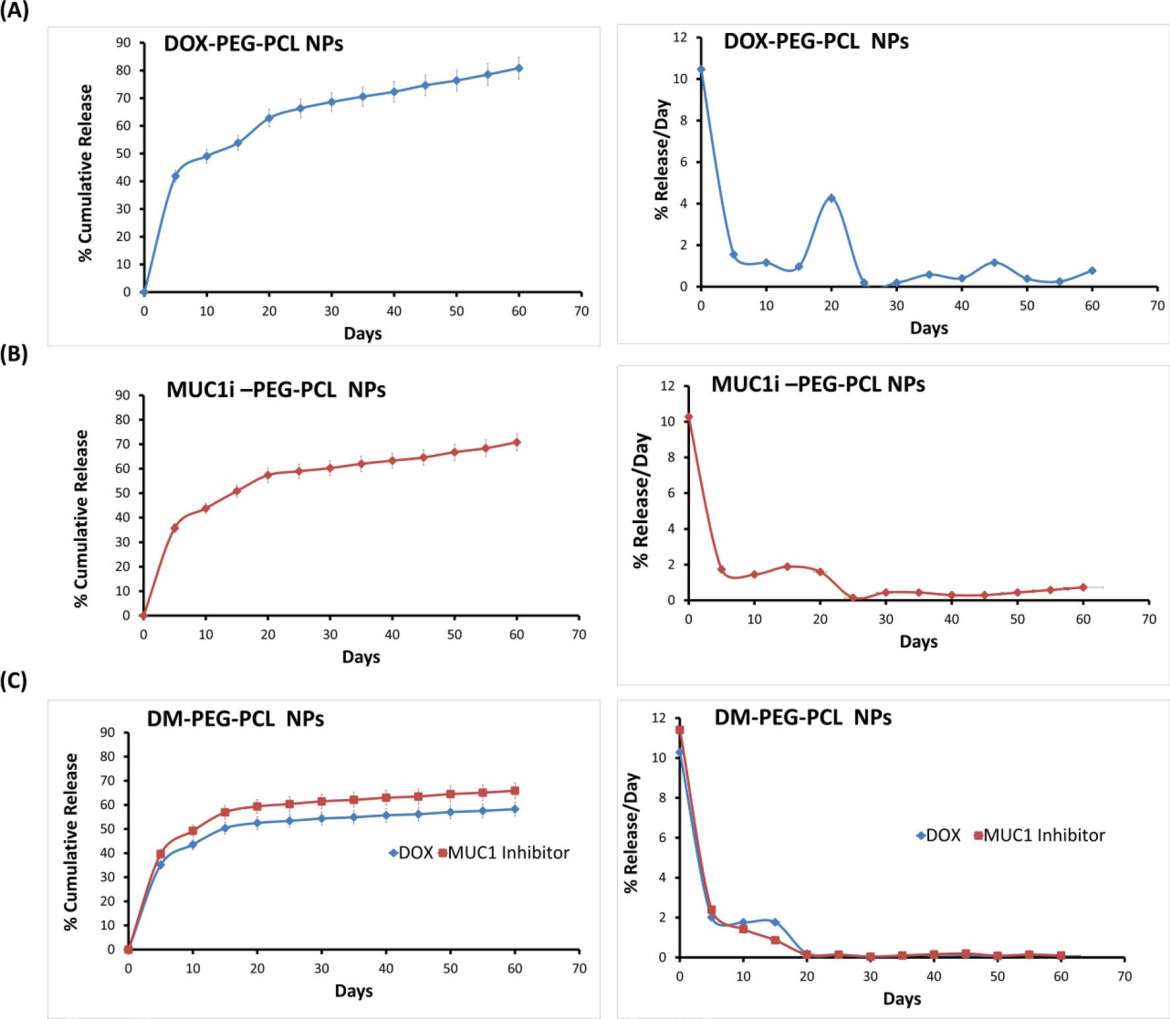
DOX and MUC1 inhibitor are released from NPs In vitro. **A** DOX release from DOX-PEG-PCL NPs. **B** MUC1 inhibitor release from MUC1i-PEG-PCL NPs. **C** DOX (circles) and MUC1inhibitor (triangles) released from DM-PEG-PCL NPs.The left panel shows % cumulative release, and the right panel shows release per day in PBS at pH 7.4 (mean ± SD of 3 repetitions)

At pH of 7.4, the In vitro release kinetics of DOX and MUC1 inhibitors from the generated NPs were evaluated. Over ten days, the cumulative release of DOX was found to be ~ 49% from DOX-PEG-PCL NPs (Fig. 2A). The burst rate of epitomized DOX released per day was initially ~ 10%, followed by a consistent release of ~ 1–2.5% per day, over two months (Fig. 1B). The rate of MUC1 inhibitor released per day (Fig. 2B) from MUC1i-PEG-PCL NPs was similar to that of DOX-PEG-PCL NPs. For 10 days, the co-release of DOX and MUC1 inhibitor from DM-PEG-PCL NPs were 43% and 49%, respectively (Fig. 2C). In addition, the release of DOX and MUC1 inhibitor from the DM-PEG-PCL NPs over 60 days was 58% and 65%, respectively (Fig. 2C).

Furthermore, the rate of DOX released per day from the DM-PEG-PCL NPs and DOX-PEG-PCL NPs was similar (Fig. 2C), while the release of MUC1 inhibitor from DM-PEG-PCL NPs was delayed compared to the MUC1 inhibitor released per day from MUC1i-PEG-PCL NPs. These results indicated that nanoparticles produced from amphiphilic block co-polymers like PEG–PCL could sustain DOX and MUC1 inhibitor release for up to 60 days. By enhancing the drug release at the site of action, stimuli-sensitive “smart” NPs, such as controlled drug release formulations, might enhance therapeutic effectiveness [41]. The sustained release enables a specific drug to be administered at a specified frequency over an extended period while minimizing its adverse effects. This approach of drug delivery is especially beneficial for pharmaceuticals that are metabolized too rapidly and expelled from the body too rapidly [42]. It has been reported earlier that nano-sized PEG-PCL drug delivery vehicles facilitate drug accumulation at tumor site, intra-tumoral diffusion, and efficient cellular uptake of the drugs, leading to improved breast cancer treatments [43]. Furthermore, it has been shown earlier that the PEG-PCL core degrades in acidic circumstances (pH 5.5) as a result of the hydrolysis of the ester bonds in the polymer chains [44]. Additionally, the acid-triggered release of the drug is aided by the dissociation of the hydrophilic DOX-MUC1i from the hydrophobic PEG-PCL core as the pH falls [45]. These studies suggest that once PEG-PCL NPs are internalized into a tumor, the acidic milieu is anticipated to enhance the effective release of the DOX-MUC1i.

The hyaluronic acid used in hydrogels is another significant drug delivery system. In the early 1960s, hydrogels have been proposed as innovative drug delivery strategies. Cross-linked polymers containing hydrophilic groups give rise to hydrogels, which can absorb a lot of water. Even though hydrogels are excellent delivery systems, they have some drawbacks that PEG-PCL nanoformulations can overcome. These drawbacks include non-biocompatible and non-biodegradable characteristics of the hydrogels, which are rebutted by PEG-PCL NPs, which are biodegradable and biocompatible. Other drawbacks of hydrogels include abrupt drug release during hydrogel swelling and large porous hydrogels. In contrast, PEG-PCL NPs had a better drug loading and sustained drug release [46]. The current research work is a novel synergistic approach involving concomitant loading of DOX with the MUC1 inhibitor in PEG-PCL polymer for the treatment of triple-negative breast cancer.

### Intracellular Drug Absorption and Cell Viability Effects of DOX and MUC1 Inhibitor Loaded NPs

**Fig. 3:**
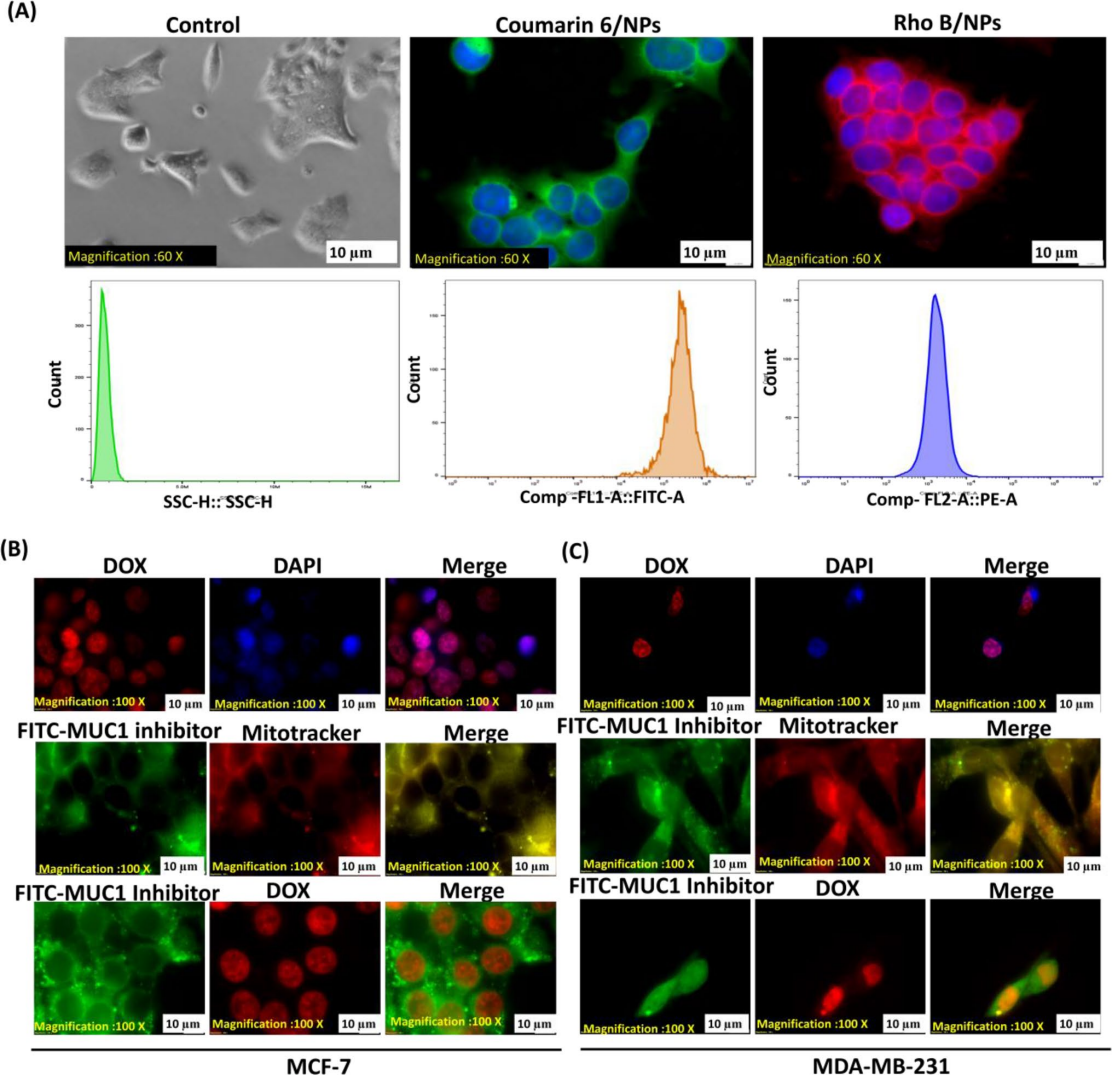
Studies on cellular absorption and the impact of NPs on DOX and MUC1 inhibitor intracellular localization. **A** Cellular uptake of NPs loaded with the RhoB or C6 dyes on MCF-7 for 12 h. Assessed by fluorescence microscopy (60X magnification) and FACS. MCF-7 (**B**) and MDA-MB-231 (**C**) Fluorescence microscopy(100X magnification) images of DAPI and DOX co-localization, in the upper panel; MUC1 inhibitor and mitotracker co-localize in a yellow/orange signal, in the middle panel, and MUC1 inhibitor is shown in green with DOX depicting red signal

To investigate cellular internalization and intracellular trafficking, polymeric PEG-PCL NPs were loaded with hydrophobic Rhodamine B (RhoB) and hydrophilic Coumarin-6 dyes. The dye-labeled NPs were then analyzed for their cellular uptake by FACS analysis as well as fluorescence microscopy. Figure 2A confirms the intracellular localization of these dye-labeled PEG-PCL NPs in MCF-7 cells. Additionally, after 12 h of incubation, DOX-PEG-PCL NPs were found to be localized in the nucleus of both MCF-7 and MDA-MB-231 cells (Fig. 3B and C). The difference in spectrum shape and fluorescence intensity between free and DNA-attached doxorubicin was assessed in fluorescence intensity spectra from a micro-volume of single decent cell nuclei administered with the drug.

**Fig. 4:**
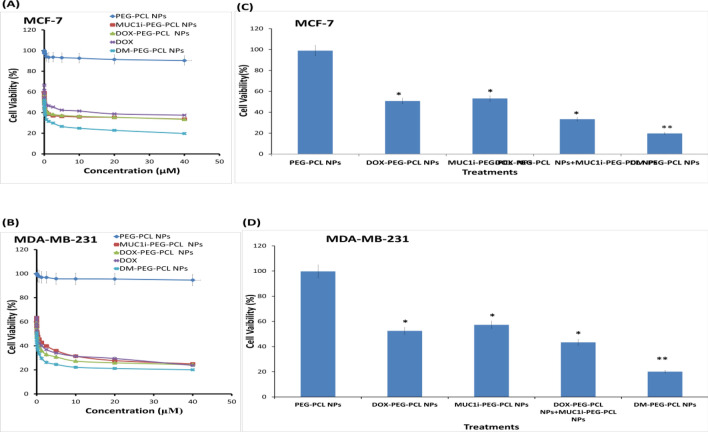
Impact of NPs on DOX and MUC1 inhibitor intracellular location and cell viability. MCF-7 (**A**) and MDA-MB-231 (**B**) cells were treated at 1:1 ratios with NPs entrapped with DOX and MUC1 inhibitor.MTT tests were used to examine the cells after 48 h. The % viability (mean ± SD of three separate trials) is provided. MCF-7 (**A**) and MDA-MB-231 (**B**) cells were given 48 h of treatment with PEG-PCL NPs, DOX-PEG-PCL NPs, MUC1i-PEG-PCL NPs, and DM-PEG-PCL NPs. Cell viability was determined using MTT assay. The % viability (mean ± SD of three separate trials) is provided. MCF-7 (**C**) and MDA-MB-231 (**D**) cells were treated for 72 h with a 0.01 µM concentration each. MTT tests were used to evaluate cell viability. The results are given as % of viable cells. (mean ± SD of three replicates)

Furthermore, mitotracker labeling revealed that the MUC1i-PEG-PCL NPs were restricted to the cytoplasm and mitochondria (Fig. 3B and C) [47]. Fluorescence analysis of MCF-7 and MDA-MB-231 cells treated with FITC- and DOX-PEG-PCL NPs revealed that DOX was localized in the nucleus and MUC1 inhibitor in the mitochondria. Most human carcinomas have abnormally high levels of the MUC1 heterodimer trans-membrane glycoprotein. The MUC1 C-terminal subunit is found in mitochondria and prevents the intrinsic apoptotic pathway from activating stress [48]. MCF-7 and MDA-MB-231 cells were treated with DOX-PEG-PCL, MUC1i-PEG-PCL, and DM-PEG-PCL NPs to determine the effect of NPs on cell viability. PEG-PCL NPs did not show any significant cytotoxicity up to 40 µM concentration even after 48 h of incubation, confirming that the NPs act purely as vehicles for the drug. Any observed cytotoxicity of the drug entrapped NPs will thus be mainly accredited to the effects of the released drugs from the nanoparticles. As revealed in Fig. 4, the cytotoxicity of free DOX, DOX-PEG-PCL NPs, MUC1i-PEG-PCL NPs, and DM-PEG-PCL NPs were dose-dependent. Compared to DOX-PEG-PCL NPs and MUC1i-PEG-PCL NPs, the DM-PEG-PCL NPs showed higher cytotoxicity against breast cancer cells as depicted by their IC_50_ values (Table 2). The enhanced cytotoxic activities of DM-PEG-PCL NPs could be ascribed to the synergistic effects of DOX and MUC1 inhibitor in cancer cells. Earlier, the paclitaxel-(MUC1) aptamer-modified PEG-AuNPs have shown In vitro cytotoxicity towards MUC1-positive breast cancer cells [49].

**Table 2:** IC_50_ values of the synthesized nanoformulations in breast cancer cell lines MCF-7 and MDA-MB-231 cells

Entry	Treatment	IC_50_ values (nM)	
		MCF7 MDA-MB-231	
1	DOX	104	133
2	DOX-PEG-PCL NPs	25.7	38
3	MUC1i-PEG-PCL NPs	44.3	133.7
4	DOX-PEG-PCL NPs + MUC1i-PEG-PCL NPs	44.8	105.4
5	DM-PEG-PCL NPs	5.8	2.4

According to a previous study, PEG-based NPs gain entry in the cells through endocytosis, which was associated with surface charge flipping (anionic to cationic) at the acidic pH of endo-lysosomes [50–52]. This charge flipping of the NPs help them to engage with vesicular membranes, leading to the NPs release into the cytosol [53]. The increased permeability and retention (EPR) effect permits nanoparticles to enter the body’s rapidly dividing cells. Because tumor cells divide at the highest rate, their uptake of NPs is massively greater [54]. DOX is autofluorescent and have been shown to swiftly penetrate cells via passive diffusion [55].

In the near future, combination therapy, a therapeutic approach that combines two or more medicinal drugs, will be a key strategy to fight against diseases like cancer, COVID-19. Instead of utilizing monotherapy, doctors are increasingly prescribing many drugs at once to increase therapeutic effectiveness since this strategy may target important pathways in a synergistic fashion [56]. To check whether the combination of DOX and MUC1i in cocktail nanoparticles is showing any synergy, we took a physical mixture of DOX-PEG-PCL NPs and MUC1i-PEG-PCL NPs, and analyzed the effect of these nanoparticles on MCF-7 and MDA-MB-231 cell lines. The data suggested that MCF-7 and MDA-MB-231 cells administered by the physical mixture of DOX-PEG-PCL NPs and MUC1i-PEG-PCL did not show any combination effect (Table S1). On the other hand, co-delivered DOX and MUC1 inhibitor entrapped jointly in PEG-PCL NPs displayed a synergistic effect on breast cancer cell death, giving much lower IC_50_ values (Table S1). The synergetic inhibition was seen in both the cell lines.

The Chou and Talalay analysis was used to calculate the combination Index (CI) by Compusyn software [28]. The results demonstrated synergy between DOX and MUC1 inhibitor with CI values < 1.0 (Fig. 4C and D) in MCF-7 and MDA-MB-231 cell lines. Tables S1 and S2 show that DM-PEG-PCL NPs suppressed breast cancer cell proliferation and survival in a synergistic way. One important method for treating tumors that are resistant to therapy is the use of combination medicines. For instance, chemotherapeutics and powerful efflux transporter inhibitors can be used to improve the exposure of cancer cells to the deadly medication and hence restore therapeutic benefits [57, 58].

### Effect of NPs on Mitochondrial Membrane Potential (MMP)

**Fig. 5:**
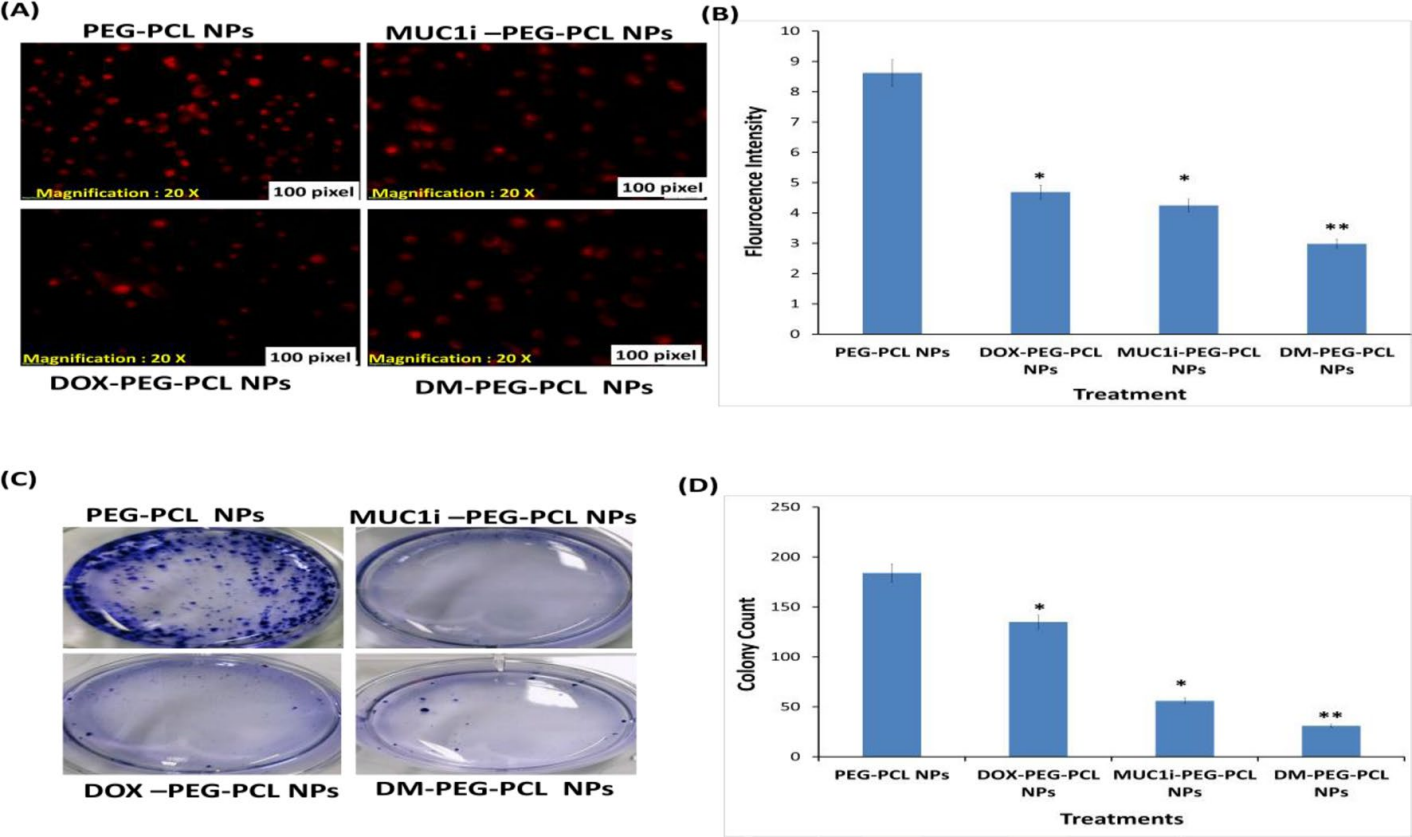
Reduction in mitochondrial membrane potential **A** MCF-7 cells were treated with DM-PEG-PCL NPs at 5.8 nM, DOX-PEG-PCL NPs at 25.7 nM, and MUC1i-PEG-PCL NPs at 43.3 nM alone. The Rh 123 fluorescence signals decreased considerably in correlation with the reduction in mitochondrial membrane potential, according to the findings (60X magnification). **B** The intensity of fluorescence in all of the treatments and the control is represented graphically. Values are presented as mean ± SD (*n* = 3), * represents *p* < 0.05, ** represents *p* < 0.01 and *** represents *p* < 0.001. Reduction in colonies in treatment **C** Representative images of colony development on MCF-7 cells after two weeks of various treatments DOX-PEG-PCL NPs at 25.7 nM, MUCi-PEG-PCL NPs at 43.3 nM, DM-PEG-PCL NPs at 5.8 nM, and PEG-PCL NPs as control. (D) Statistic data of various treatments. (*, *p* < 0.05, **, *p* < 0.01)

The mitochondria are the cell’s power plants since they transform oxygen and nutrients into adenosine triphosphate. In mammalian cells, mitochondrial membrane depolarization is the main event that irreversibly commits a cell to die. Bcl-2 family proteins induce the release of proteins from the mitochondrial intermembrane, leading to the execution of apoptosis [59, 60]. Rhodamine 123 (Rh 123) is a cationic probe that can be quickly internalized and incorporated inside the mitochondria of a living cell [61]. A decrease in mitochondrial membrane potential [62] marks early apoptosis. The observed data revealed that DOX-PEG-PCL NPs, MUC1i-PEG-PCL NPs, and DM-PEG-PCL NPs at 25.7 nM, 44.3 nM, and 5.8 nM concentrations, respectively, caused mitochondrial perturbation after incubating for 48 h (Fig. 5A). DOX-PEG-PCL NPs and MUC1i-PEG-PCL NPs caused a ~ two-fold reduction in Rh 123 fluorescence in MCF-7 cells, while DM- PEG-PCL NPs treatment led to 2.7-fold reductions in fluorescence (Fig. 5). These results clearly showed that DM-PEG-PCL NPs mediated mitochondrial depolarisation in MCF-7 cells leading to cancer cell death.

The mitochondria are the key player in apoptosis induction. The truncated BID (pro-apoptotic protein) induces oligomerization of BAK on the mitochondrial surface where it triggers release of cytochrome c leading mitochondrial membrane permeabilization. This event is followed by activation of caspase-3 and hence apoptosis [63]. Many environmental pollutants cause mitochondrial injuries via release of pro-apoptotic genes into the cytoplasm and initiate apoptosis in an oxidative stress-related mitochondrial pathway. As a result, mitochondria-targeted antioxidants are increasingly being developed to prevent mitochondrial oxidative damage and ROS production. These antioxidants are also becoming widely available as over-the-counter supplements. Contrary to general antioxidants, mitochondria-targeted antioxidants are chemically altered to allow them to pass cellular membranes where they accumulate and reduce ROS levels [64]. For instance, the study conducted by Ahmadian et al., showed that quercetin-loaded nanostructured lipid carriers (QNLC) effectively reduced paraquat-mediated mitochondrial toxicity. The study also revealed a reduction in ROS levels and paraquat induced cell death by QNLC in human lymphocytes. Therefore, QNLC could be a promising antioxidant carrying drug delivery system for counteracting paraquat-mediated toxicity [65].

### Colony Formation

The colony formation experiments were utilized to see how our DM-PEG-PCL NPs impacted MCF-7 cell growth over time. MCF-7 cells were cultured for two weeks with treatment groups DOX-PEG-PCL NPs, MUC1i-PEG-PCL NPs, and DM-PEG-PCL NPs with IC_50_ values of 25.7 nM, 43.3 nM, and 5.8 nM, respectively, in six-well plates (Fig. 5A). The colony formation was statistically reduced in the presence of DM-PEG-PCL NPs (83%), whereas treatment using DOX-PEG-PCL NPs and MUC1i-PEG-PCL NPs led to 26% and 70% reduction in colony formation, respectively (Fig. 5C). DM-PEG-PCL NPs reduced colony formation in MCF-7 cell lines, validating cancer cell death. This indicated that cocktail-loaded NPs had a considerably stronger anti-proliferative effect than single-drug-loaded nanoparticles (DOX-PEG-PCL NPs and MUC1i-PEG-PCL NPs).

### Inhibition of Cell Migration

**Fig. 6:**
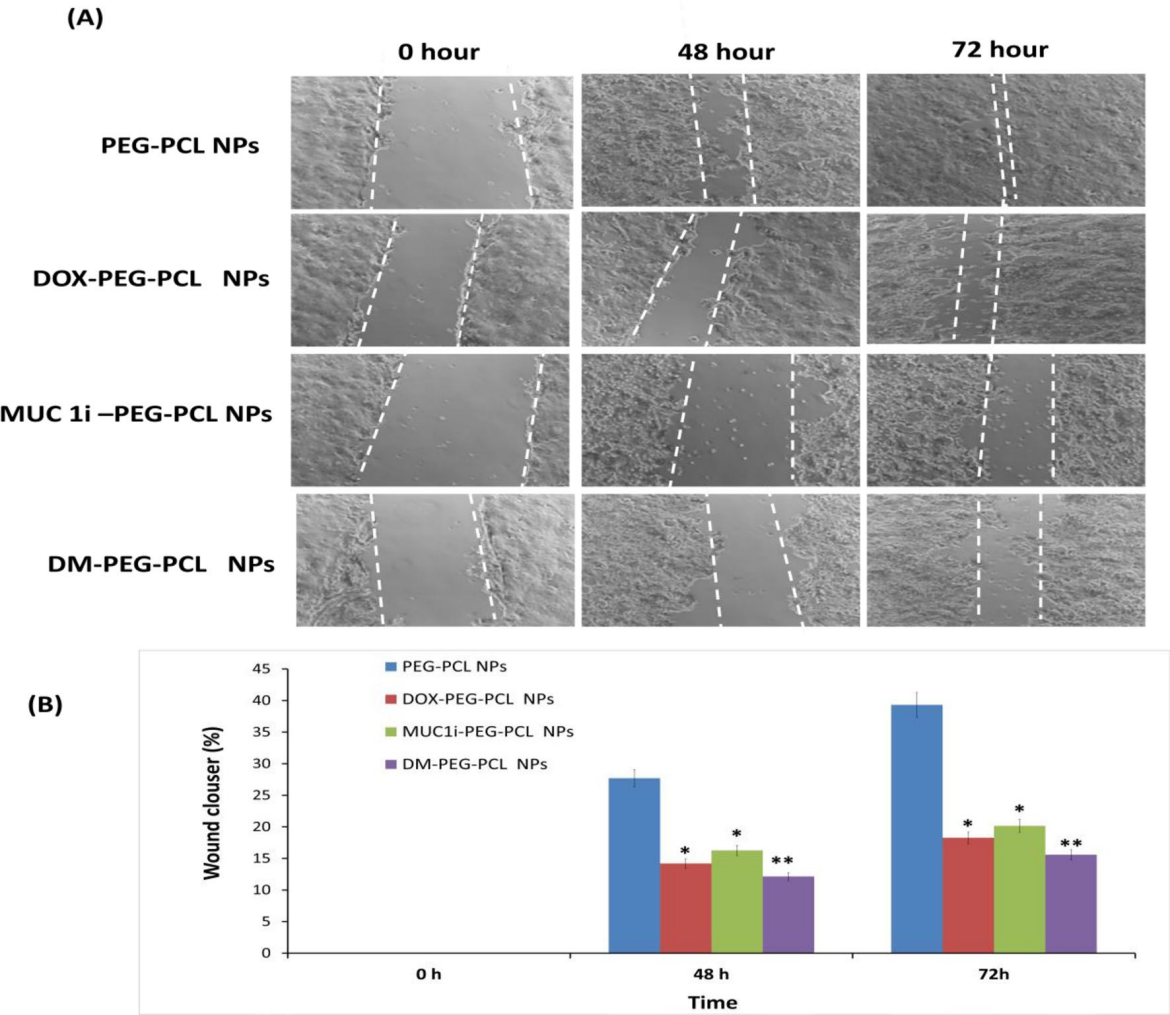
Wound healing assay **A** illustrates the migration of MCF-7 cells at 0 h, 48 h, and 72 h. DOX-PEG-PCL NPs at 25.7 nM, MUCi-PEG-PCL NPs at 43.3 nM, DM-PEG-PCL NPs at 5.8 nM, and PEG-PCL NPs as control were used at different times. The therapy stops the cells from migrating. Scratched and recovering injured regions (indicated by white lines) on MCF7 cell confluence monolayer at various periods following treatment. **B** Wound closure semi-quantitative analysis. (*p < 0.05, **p < 0.01)

The wound-healing tests were used to investigate the functional impact of these NPs on cancer cell migration. Compared to the untreated control (PEG-PCL NPs) for 48 and 72 h, DM-PEG-PCL NPs substantially suppressed the migration of MCF-7 cells (Fig. 6A, B). The percentage of wound closure using DM-PEG-PCL NPs was observed to be around 12.1% and 15.5%, respectively, after 48 and 72 h of incubation, whereas treatment using DOX-PEG-PCL NPs and MUC1i-PEG-PCL NPs caused 18.2% and 20.1% gap closure, respectively. PEG-PCL NPs displayed higher gap closure of 39.3% even after 72 h of incubation (Fig. 6). This data clearly illustrates that our developed DM-PEG-PCL NPs can inhibit cancer cell growth compared to blank NPs. Compared with single drug-loaded nanoparticles (DOX-PEG-PCL NPs and MUC1i-PEG-PCL NPs), cocktail DM-PEG-PCL NPs exhibited the strongest inhibition effect on both proliferation and invasion of MCF-7 cells, which reflected the synergistic efficacy of MUC1 inhibitor and DOX towards breast cancer cells.

### Evaluation of Apoptosis Caused by DM-PEG-PCL NPs

**Fig. 7:**
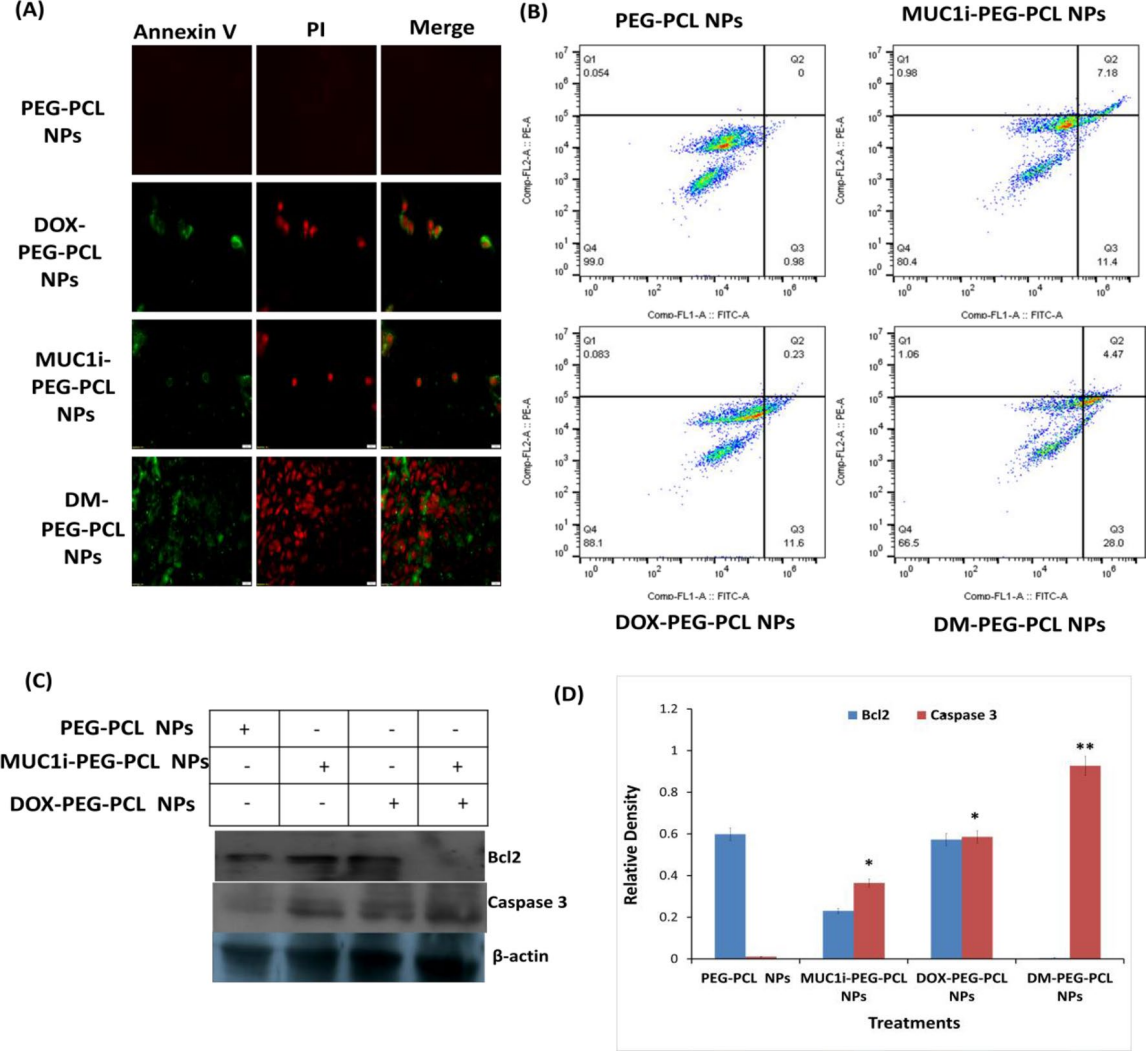
Effects of NPs on the induction of apoptosis. **A** Fluorescence microscopy images **B** FACS of Annexin V/PI double staining in MCF-7 cells treated for 48 h with PCL NPs (negative control), MUC1i-PEG-PCL NPs at 43.3 nM, DOX-PEG-PCL NPs at 25.7 nM, and DM-PEG-PCL NPs at 5.8 nM. (BD Biosciences, San Jose, California).**C** Immunoblotting **D** quantify immunoblots for 48 h, MCF-7 cells were given PEG-PCL NPs (negative control), MUC1i-PEG-PCL NPs at 50 nM, DOX-PEG-PCL NPs at 31.4 nM, and DM-PEG-PCL NPs at 11.5 nM. Immunoblotting with the identified antibodies was used to examine whole cell lysates and quantification through Image J software

Phosphatidylserine (PS) residues can be found in the cytoplasmic membrane's inner membrane in normal cells. PS residues are translocated to the outer surface of the membrane during apoptosis which also acts as a signal to neighbouring cells that a cell is ready to be phagocytosed [66]. A PS-binding protein, Annexin V, has been utilized to identify apoptotic cells. Annexin V/Propidium Iodide (PI) staining was performed to analyze nanoparticle-mediated apoptosis induction in MCF-7 cells for 30 min of incubation. As depicted in Fig. 7A, the Annexin V/PI staining was not evident in untreated cells, while MCF-7 cells treated with DM-PEG-PCL NPs showed significant Annexin V/PI staining, thus signifying apoptosis in these cells [67]. To support this data, further experiments using flow cytometry were performed using DM-PEG-PCL NPs, DOX-PEG-PCL NPs and MUC1i-PEG-PCL NPs in MCF-7 cells (Fig. 7B). It was seen that 28% of DM-PEG-PCL NPs treated cells were going to early apoptosis compared to DOX-PEG-PCL NPs and MUC1i-PEG-PCL NPs, (11% and 11.4%, respectively). It was also observed that DOX and MUC1 inhibitor promotes late apoptotic/necrotic response, as depicted by annexin V/PI staining. These data suggest that DM-PG-PCL NPs can accelerate both apoptotic and necrotic cell death.

Caspase-3 is one of the most crucial apoptosis effectors, and its activation signals produce irreversible cell death [68]. Furthermore, the B-cell lymphoma 2 (Bcl-2) family proteins, which include anti-apoptotic and pro-apoptotic members, are the most well-studied protein families for regulating apoptotic cell death [69]. The western blot analysis revealed a reduction in Bcl-2 (~ fivefold reduction) and enhancement in caspase-3 (~ threefold increase) expression using DM-PEG-PCL NPs at an IC_50_ value of 11.5 nM on MCF-7 cells compared to the blank NPs.

These findings supported the idea that DOX and MUC 1 inhibitor co-delivery via DM-PEG-PCL NPs could activate the cell death pathway in MCF-7 cells by increasing caspase-3 and reduced Bcl-2 expression. MUC1-C is also expressed in mitochondria's outer membrane, where it protects mitochondrial transmembrane potential and inhibit intrinsic apoptotic pathway [70]. MUC1-C interact with pro-apoptotic BAX (Bcl-2 Associated X, Apoptosis Regulator) protein's BH3 domain and blocks BAX function [71]. The interaction between MUC1-C and BAX is disrupted in breast cancer cells in the presence of MUC1 inhibitor [71].

This increase in caspase-3 expression and downregulation of Bcl-2 expression in DM-PEG-PCL NPs treated breast cancer cells suggest mitochondrial disruption and apoptosis induction. Apoptosis refers to a coordinated and energy-dependent cell death process that is essential for tissue survival and homeostasis. Morphological indicators of apoptosis include membrane blebbing, nuclear condensation, and the development of apoptotic bodies. Additionally, the fragmentation of chromatin into tiny pieces is a physiological indicator of apoptosis [72]. The citalopram, an antidepressant has been shown to induce apoptosis in liver hepatocellular carcinoma cell line HepG2. Citalopram treatment led to enhanced ROS and mitochondrial Bax levels and a decrease in Bcl2 levels in cancer cells thereby causing cytochrome c release [73].

### In Vivo Acute Toxicity Studies

As per OECD guidelines, we conducted acute toxicity studies. According to OECD guidelines, acute toxicity studies are carried out to determine the LD50 values, which are then used to determine the safe dosage range during which the drug may be administered to an animal without endangering or killing it. The trial lasted 14 days, and the dose range was from a low of 5 mg/kg to a high of 2000 mg/kg [36]. No fatality was observed in acute toxicity trials, indicating that all nanoformulations (DM-PEG-PCL NPs, DOX-PEG-PCL NPs, and MUC1i-PEG-PCL NPs) were safe even at a high dose of 2,000 mg/kg. There were no unanticipated changes in behavior, motor function, and no vertigo or intoxicated symptoms were observed over 14 days. Furthermore, no differences in growth between the animals fed with different nanoparticles and the control group were observed (Table S3). Additionally, no alterations in the fur coat, eyes, or respiratory processes were evident. The treatment and control groups did not differ significantly in terms of food and water intake. Hematological parameters of both treated and control animals showed no significant differences (*p* > 0.05) (Table S4). In biochemical studies, there was no significant difference between the control and experimental groups of rats treated with nanoformulations (*p* > 0.05) (Tables S5). We concluded that these nanoformulations had no damaging consequences on organs or their functions and there was no toxicity or mortality.

The PEG, PCL, and PEG-PCL products have been approved by FDA [74–76]. PEG-PCL-based NPs within a size range of around 20 nm to 200 nm having negative zeta potentials have shown only minor toxic effects on cell viability [77]. In vivo studies have shown that intravenous injection of a high dose (2.4 g/kg) of PEG-PCL micelles did not display acute toxicity. Moreover, no histological changes in the heart, lung, liver, kidney, and spleen were observed [78]. Furthermore, PEG-b-PCL micelles have been tested for sub-chronic toxicity (i.v., 100 mg/kg daily for one week) in rats and mice. The treatment did not cause any weight gain and no toxic or acute inflammation on the liver, kidney, or brain parenchyma was observed [79, 80] Therefore, all these earlier published reports and our results depict that PEG-PCL-based nanoformulations do not induce any toxicity and are safe in experimental animals.

### In Vivo Anti-Cancer Activity of Our Developed Nanoformulations

**Fig. 8:**
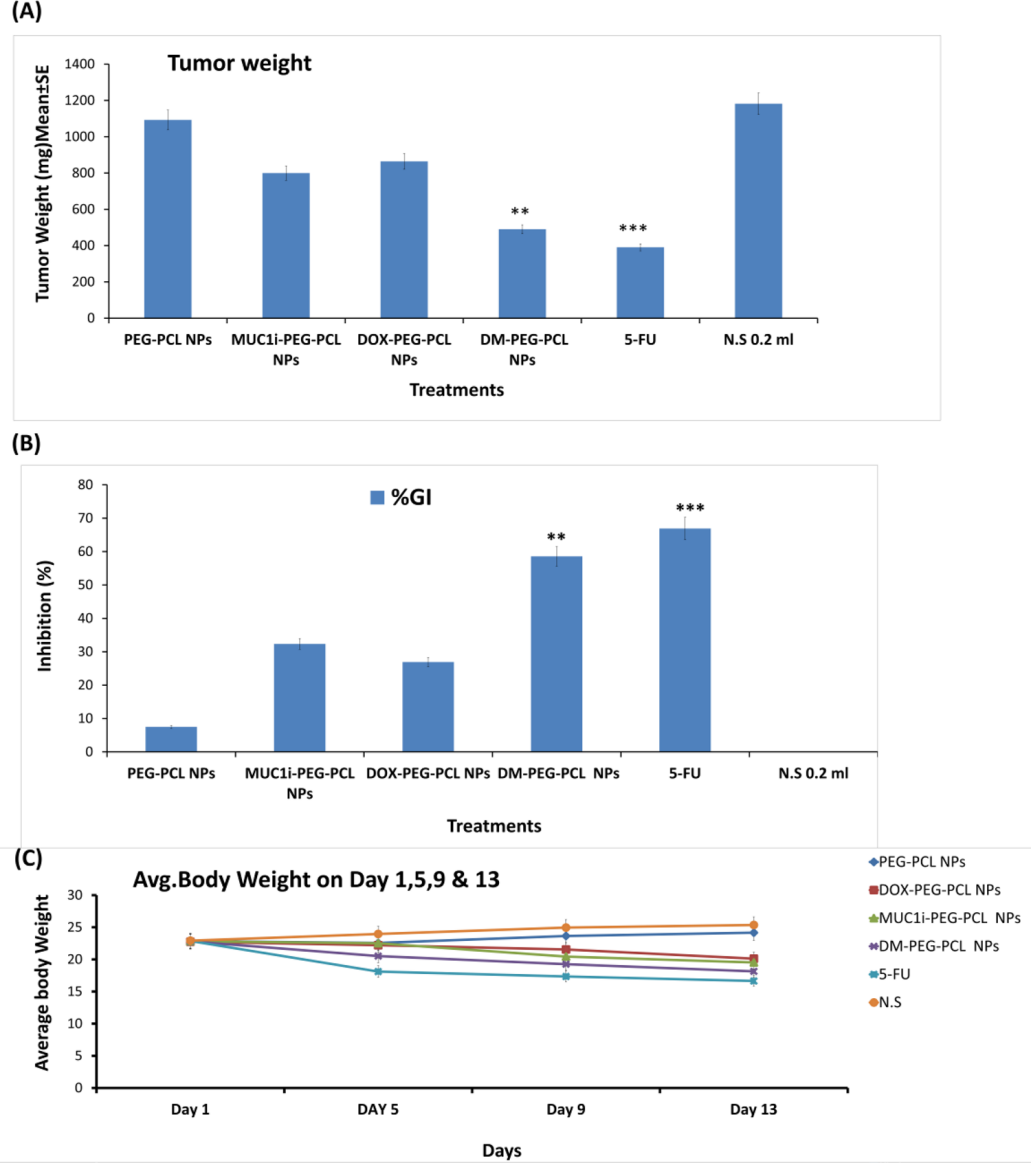
*In-vivo anticancer activity of* DOX-PEG-PCL NPs, MUC1i-PEG-PCL NPs, and DM-PEG-PCL NPs in EAT model. The mice were administered treatments (10 mg/kg) intraperitoneally for 9 days after the cancer cells were implanted (i.m.), and the tumors were assessed on day 13. DOX-PEG-PCL NPs, MUC1i-PEG-PCL NPs, and DM-PEG-PCL NPs were given to treated mice, whereas vehicle was given to control animals. The use of 5FU as a positive control was used. **A** Tumor weight after various treatments. **B** Growth inhibition percentage and mortality after various treatments. **C** Average body weight after various treatments. Data are mean ± SE (*n* = 7) and One-way comparisons ANOVA were conducted between the control and treated groups. p -values *p < 0.05, **p < 0.01and***p < 0.01 were considered significant

EAT-bearing Swiss albino mice have been used to assess the anti-cancer capability of DM-PEG-PCL NPs, DOX-PEG-PCL NPs, and MUC1i-PEG-PCL NPs. The solid tumor was induced by injecting EAC intramuscularly and NPs (10 mg/kg body weight) were administered intraperitoneally for nine days after the EACs inoculation. In comparison to the untreated and empty NPs (1181.8 and 1092.28 mm^3^), EAT-bearing mice treated with DM-PEG-PCL NPs and 5-Fluorouracil (5-FU) showed a considerable reduction in tumor size (489.9 and 390.4 mm^3^). In contrast to the untreated control, DOX-PEG-PCL NPs and MUC1i-PEG-PCL NPs (863.9 and 799.5 mm^3^) therapy reduced tumor growth. Furthermore, DM-PEG-PCL NPs and 5-FU demonstrated a substantial tumor inhibition of 55.5 and 66.9%, respectively, compared to untreated or empty NPs treated mice (Fig. 8A). On the other hand, the DOX-PEG-PCL NPs, and MUC1i-PEG-PCL NPs displayed only 26.9 and 32.34% tumor growth inhibition. Mortality was not observed in DM-PEG-PCL NPs, MUC1i-PEG-PCL NPs, DOX-PEG-PCL NPs, and 5-FU treated EAT-bearing mice. Hence, DM-PEG-PCL NPs showed better tumor inhibition than DOX-PEG-PCL NPs and MUC1i-PEG-PCL NPs alone. Additionally, the sustained release of DOX and MUC1 inhibitors could contribute to the tumor inhibition capabilities of these developed nanoparticles [81, 82].

This data suggest that synergistic effect of DOX and MUC1i suppressed tumor growth in an in vivo model as well. As shown above, DM-PEG-PCL NPs showed sustained release over sixty days, which is critical for an effective drug delivery mechanism. Finally, increased cellular uptake, enhanced doxorubicin retention, and cancer cell death induction, make this nanoformulation a promising anticancer agent.

Furthermore, there have been other novel biomaterials employed for the drug delivery. The small-molecule exogenous antioxidants can serve as therapeutic agents for several oxidative damage bases pathologies, still these molecules have not been successful. A good exogenous antioxidant should be easily absorbed and transported to the intracellular region so that it can modulate gene expression for preventing pathological oxidative damage. Therefore, the need of the hour is to develop novel nanoscale drug delivery methods to build effective antioxidant medicines [83]. One of the most promising nanocarriers being produced are polymeric nanoparticles, which are primarily made of synthetic biodegradable polymers. An aqueous extract of *Syzygium cumini* (ASc) seeds was combined with one of the well-known synthetic polymers recognised by the US-FDA, PCL, using an emulsification/evaporation solvent method. The data showed that even at a relatively low concentration (100 g/mL), both ASc and PCL-ASc exhibit significant DPPH radical scavenging activity as well as reducing power in the FRAP assay, indicating that the immobilisation of ASc in PCL nanoparticles had no effect on this activity [84]. In order to deal with the problems with encapsulation process, such as initial burst release, instability, and incomplete release, as well as targeted drug delivery, Nayak et al. prepared chitosan nanoformulations, encapsulating antioxidants such as ascorbic acid (vitamin C), tochopherol (vitamin E), and catechol [85]. These nanoantioxidants showed better free radicals scavenging potential than their base materials and effective anticancer activity towards breast cancer cells. Doxorubicin is also delivered to the target tissues using a different technique employing single-walled carbon nanotubes (SWCNT). To increase the SWCNTs' biocompatibility and solubility, chitosan/folic acid was used to wrap them [86].

Biodegradable PEG-PCL nanoparticles are widely used for drug delivery system. Rui et al., developed PCL-PEG copolymer-based nanoparticles (PCL-PEG-Tyr/Ang) by using tyrosine (Tyr) and angiopep-2 (Ang) as coupling ligands for the intravenous delivery of docetaxel. When compared to other PCL-PEG-based nanoparticles, the dual-modified PCL-PEG-Tyr/Ang nanoparticles showed enhanced cytotoxicity towards HT29 colorectal cancer cells. According to the in vivo imaging, PCL-PEG-Tyr/Ang nanoparticles were more effective at targeting tumors than other PCL-PEG-based nanoparticles. Docetaxel-loaded PCL-PEG-Tyr/Ang nanoparticles demonstrated a stronger inhibitory efficacy on tumor growth than the Taxotere®-treated HT29 tumor-xenografted nude mice [87]. In another recent study, the PEG-PCL copolymer was encapsulated with curcumin (CUR) as a therapeutic anticancer drug and conjugated to folic acid utilizing lysine as a linker. MTT assay and hemolysis assay showed anticancer potential on breast cancer cells and biocompatibility of these nanocarriers, respectively. In mouse model, these nanocarriers did not cause substantial weight loss or side effects suggesting that CUR loaded nanocarriers conjugated with folic acid could act as targeted anticancer agent [88]. In another study, BTN-PEG-PCL and mono methoxy PEG-PCL (mPEG-PCL) diblock co-polymers series were created and their in vitro and in ovo toxicity was assessed. The CUR loaded BTN-PEG-PCL enhanced sub-G1 cell population and apoptosis in cancer cell lines. Additionally, an in ovo experiment on chick chorioallantoic membrane showed that CUR-BTN-PEG-PCL significantly reduced tumor cell growth and angiogenesis [89]. A recent report showed that codelivery of gemcitabine and MUC1 inhibitor using PEG-PCL nanoparticles significantly enhanced MCF-7 breast cancer cells killing. Also, substantial decrease in tumor size was observed in the Ehrlich ascites tumor-bearing mice [90].

## Conclusion

The current study deals with the synthesis of PEG-PCL copolymer. These copolymers were then self-assembled into nanoparticles by double emulsion method with entrapment of both DOX and MUC1 inhibitor (GO-201). The spherical-shaped DM-PEG-PCL NPs showed synergistic effects of DOX and MUC1 inhibitor In vitro, as revealed by their lower IC_50_ values compared to free DOX, MUC1i, and other controls. In vivo experiments with DM-PEG-PCL NPs in the EAC murine model revealed considerable inhibition in tumor growth compared to DOX or MUC1i encapsulated NPs. Furthermore, acute toxicity profiles confirmed the non-toxic behavior of the DM-PEG-PCL NPs with no adverse effect on organs and their functions along with no mortality. The drug release profiles from the synthesized NPs were found to be sustainable over 60 days. Pharmacokinetic studies can be performed in the future to understand drug absorption, distribution, metabolism, and clearance of the prepared nanoformulation from the animal body. Furthermore, the efficacy of the nanocomposite on patients’ tumor tissues can also be analyzed. Also, PEG-PCL nanoparticles could act as a drug delivery system to enhance the bioavailability of the anticancer drugs. To conclude, PEG-PCL nanoparticles loaded with DOX and MUC1i show great potential as novel nanomedicine for the treatment of triple-negative breast cancer.

## Supplementary Information

Below is the link to the electronic supplementary material.

Supplementary file1 (DOCX 8100 KB)

## Abbreviations

P-GP: Poly-glycoproteins
DOX: Doxorubicin
IP: Intraperitoneal
PDI: Polydispersity index
EAT: Ehrlich ascites tumor
TEM: Transmission electron microscopy
TNBC: Triple-negative breast cancer
NP: Nanoparticle
