# Supplementary material for: Biodegradable PEG-PCL Nanoparticles for Co-delivery of MUC1 Inhibitor and Doxorubicin for the Confinement of Triple-Negative Breast Cancer
Source: J Polym Environ. 2022 Nov 11;31(3):999–1018. doi: 10.1007/s10924-022-02654-4 (PMC9651876; doi:10.1007/s10924-022-02654-4)
Supplement: Supplementary file 1 — Supplementary file1 (DOCX 8100 KB) [file 10924_2022_2654_MOESM1_ESM.docx]

Supporting Information

**Biodegradable PEG-PCL nanoparticles for co-delivery of MUC1 inhibitor and doxorubicin for the confinement of triple-negative breast cancer**

Akanksha Behl^a^, Subhash Solanki^b^, Shravan K. Paswan^c^, Tirtha K. Datta^b^,

Adesh K. Saini^d^, Reena V. Saini^d^, Virinder S. Parmar^e^, Vijay Kumar Thakur ^f-i,*^, Shashwat Malhotra^k,*^, and

Anil K. Chhillar^a,*^

*^a^Centre for Biotechnology, M.D. University, Rohtak, Haryana - 124 001, India*

*^b^Animal Biotechnology Centre, ICAR-National Dairy Research Institute, Karnal,*

*Haryana -132 001, India*

*^c^Pharmacology Division, National Botanical Research Institute ( CSIR-NBRI ), Lucknow, Uttar Pradesh – 226 001, India*

*^d^Central Research Cell and Department of Biotechnology, MMEC, Maharishi Markandeshwar Deemed University, Mullana, Ambala, Haryana – 133 207, India*

*^e^Department of Nanoscience, Graduate Center & Department of Chemistry and Environmental Science, Medgar Evers College, The City University of New York, 1638 Bedford Avenue, Brooklyn, NY 11225, USA*

*^f^ Biorefining and Advanced Materials Research Centre, Scotland's Rural College (SRUC), Kings Buildings, Edinburgh, EH9 3JG, UK*

*^g^ School of Engineering, University of Petroleum and Energy Studies (UPES), Dehradun, 248007 Uttarakhand, India*

*^h^Centre for Research and Development, Chandigarh University, Mohali, 140413 Punjab, India*

*^i^Department of Biotechnology, Graphic Era Deemed to be University, Dehradun 248002, Uttarakhand, India*

*^k^Department of Chemistry, Kirori Mal College, Delhi – 110 007, India*

**Table of content**

^1^H NMR spectrum of PEG-PCL co-polymer in CDCl_3_…………………………………………………..S3

FTIR spectrum of PEG-PCL co-polymer………………………………………………………………...S4

TEM images of MUC1 inhibitor, DOX, DOX -MUC1 inhibitor loaded PEG–PCL nanoparticles…….S4

DLS of DM-PEG-PCL NPs……………………………………………………………………..……… S5

The cell vaibilty graph on MCF10 A cell line…………………………………………………………… S5

The combination index (CI) and fraction affected (Fa) values on MCF-7 cells……………………..….S6

The combination index (CI) and fraction affected (Fa) values on the MDA-MB-231 cell line…………S6

Feed, water, body weight intake for Acute Toxicity Studies (14 days)…………………………………. S7

Hematological investigations of whole blood for

RBC, WBC, Haemoglobin, Platelet count, HCT, MCV, MCH and MCHC.. ………………................. S7

Clinical biochemistry investigations of serum for

Total Protein, Total Bilirubin, ALP, ALT, AST, Albumin, Glucose, Triglyceride, Creatinine,

Urea, Uric acid, Cholesterol and Alkaline phosphatase………………………………………………...S8


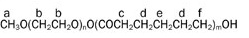


**Figure S1.** ^1^H NMR spectrum of PEG-PCL co-polymer in CDCl_3_.


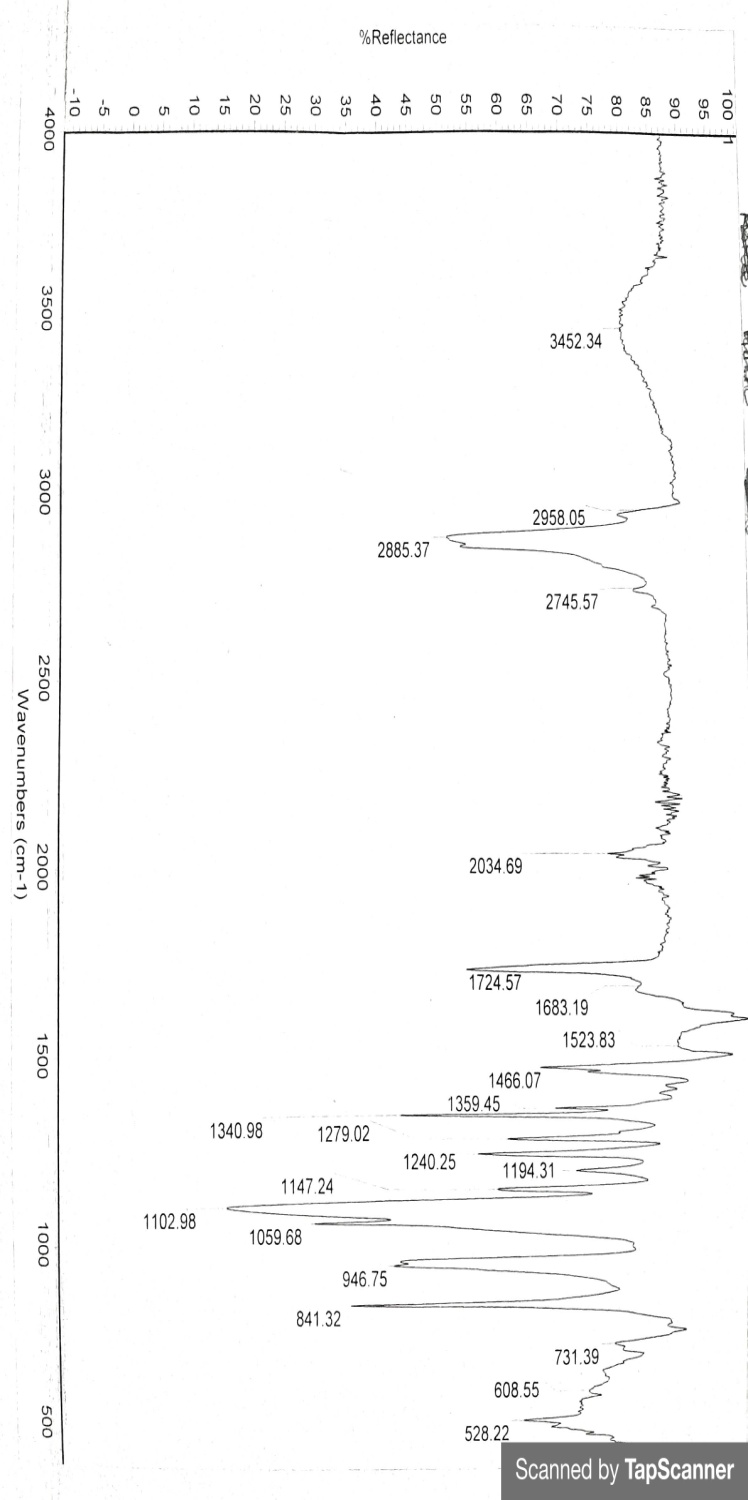


**Figure S2.** FTIR spectrum of PEG-PCL co-polymer

**Figure S3.** TEM images of MUC1 inhibitor, DOX, DOX -MUC1 inhibitor loaded PEG–PCL nanoparticles (Scale =100 nm)

**Figure S4.** DLS of DM-PEG-PCL NPs

**Figure S5.** The cell viability graph on MCF10 A (non-malignant breast epithelial cells) cell line.

**Table S1:** The combination index (CI) and fraction affected (Fa) values on MCF-7 cells.

| Comb. | DOX  (µM) | MUC1 Inhibitor  (µM) | Fa | CI Value |
| --- | --- | --- | --- | --- |
| 1 | 0.002 | 0.002 | 0.84361 | 0.0134 |
| 2 | 0.019 | 0.019 | 0.74361 | 0.0634 |
| 3 | 0.156 | 0.156 | 0.63057 | 0.0621 |
| 4 | 1.25 | 1.25 | 0.50232 | 0.06246 |
| 5 | 5.0 | 5.0 | 0.4247 | 0.07369 |
| 6 | 10.0 | 10.0 | 0.396 | 0.09299 |
|  |  |  |  |  |
| 7 | 20.0 | 20.0 | 0.3636 | 0.10923 |

**Table S2:** The combination index (CI) and fraction affected (Fa) values on the MDA-MB-231 cell line.

| Comb. | DOX  (µM) | MUC1 Inhibitor  (µM) | Fa | CI Value |
| --- | --- | --- | --- | --- |
| 1 | 0.0020 | 0.0020 | 0.15847 | 0.05514 |
| 2 | 0.019 | 0.019 | 0.14067 | 0.14066 |
| 3 | 0.156 | 0.156 | 0.1216 | 0.23991 |
| 4 | 1.25 | 1.25 | 0.0925 | 0.10948 |
| 5 | 5.0 | 5.0 | 0.07667 | 0.06445 |
| 6 | 10.0 | 10.0 | 0.0693 | 0.0466 |
| 7 | 20.0 | 20.0 | 0.06625 | 0.05938 |

**Table S3.** Feed, water, body weight intake for Acute Toxicity Studies (14 days)

|  | PEG-PCL NPs | DM-PEG-PCL NPs | DOX-PEG-PCL NPs | MUC1i-PEG-PCL NPs |
| --- | --- | --- | --- | --- |
| Body weight | 18.1±1.38 | 20.9±1.40 | 20.8±1.42 | 21.3±1.41 |
| Feed intake | 10.75±1.80 | 15.20±2.25 | 16.20±1.66 | 16.15±1.69 |
| Water intake | 10.90±1.85 | 12.30±1.95 | 12.95±1.99 | 12.35±1.90 |

Information given as mean ± SEM (n=6); p>0.05 treated groups vs control.

**Table S4.** Hematological investigations of whole blood for RBC, WBC, Haemoglobin, Platelet count, HCT, MCV, MCH and MCHC. Blood was taken from the overnight starved animals via the retro-orbital plexus.

| PARAMETERS | PEG-PCL NPs | DM-PEG-PCL NPs | DOX-PEG-PCL NPs | MUC1i-PEG-PCL NPs |
| --- | --- | --- | --- | --- |
| WBC(10^3^/uL) | 13.35±1.90 | 11.40±1.70 | 13.42±1.69 | 13.36±1.88 |
| RBC(10^6^/uL) | 7.50±0.20 | 7.021±0.043 | 7.023±0.040 | 7.48±0.22 |
| Hb (g/dL) | 14.30±0.60 | 13.15±0.09 | 13.10±0.11 | 13.31±0.58 |
| HCT (%) | 41.64±1.46 | 42.3±0.60 | 40.3±0.60 | 43.64±1.26 |
| MCV(fL) | 53.05±0.82 | 55.30±1.23 | 55.30±1.20 | 53.06±0.79 |
| MCH(pq) | 16.70±0.39 | 18.70±0.16 | 19.70±0.18 | 20.70±0.30 |
| MCHC(g/dL) | 30.8±0.56 | 31.78±0.25 | 31.79±0.22 | 32.8±0.52 |
| PLT(10^3^/uL) | 806±77.53 | 959±80.04 | 955±80.03 | 815±77.50 |

Information given as mean ± SEM (n=6); p>0.05 treated groups vs control.

**Table S5.** Clinical biochemistry investigations of serum for Total Protein, Total Bilirubin, ALP, ALT, AST, Albumin, Glucose, Triglyceride, Creatinine, Urea, Uric acid, Cholesterol and Alkaline phosphatase.

| Parameters | PEG-PCL NPs | DM-PEG-PCL NPs | DOX-PEG-PCL NPs | MUC1i-PEG-PCL NPs |
| --- | --- | --- | --- | --- |
| Glucose(mg/dl) | 107±8.93 | 103.2±10.80 | 109.1±8.92 | 109±8.90 |
| ALT(IU/L) | 51.81±2.10 | 50.33±4.99 | 50.89±2.05 | 54.81±2.03 |
| AST(IU/L) | 128.8±10.1 | 112.2±9.30 | 128.8±10.1 | 129.6±10.1 |
| ALP (IU/L) | 310.6±80.60 | 203.7±80.02 | 210.1±80.60 | 309.7±80.50 |
| Total Protein(g/dl) | 5.75±0.34 | 5.38±0.13 | 5.73±0.32 | 5.78±0.30 |
| Triglycerides(mg/dl) | 110.9±12.4 | 97.38±11.70 | 109.9±12.3 | 110.9±12.2 |
| Cholesterol(mg/dl) | 98.80±5.40 | 96.09±3.14 | 97.80±5.40 | 97.80±5.40 |
| Bilirubin (mg/dl) | 0.262±0.03 | 0.240±0.01 | 0.262±0.02 | 0.260±0.03 |
| Urea(mg/dl) | 35.25±3.06 | 40.91±2.25 | 35.2±3.12 | 35.25±3.12 |
| Creatinine (mg/dl) | 0.51±0.04 | 0.50±0.03 | 0.57±0.01 | 0.55±0.03 |

Information given as mean ± SEM (n=6); p>0.05 treated groups vs control.
